# Case Report: Renal rupture and massive urine extravasation following flexible ureteroscopic lithotripsy: analysis of 4 cases and literature review

**DOI:** 10.3389/fmed.2026.1914419

**Published:** 2026-09-29

**Authors:** Hong-Ping Tao, Gong-Hui Li, Sheng Cheng, Bin Chen, Geng Yang, Ya-Ran Chen, Xue-Ping Mao

**Affiliations:** 1 Department of Urology, The First People's Hospital of Wuyi County, Jinhua, China; 2 Department of Urology, Sir Run Run Shaw Hospital, Zhejiang University School of Medicine, Hangzhou, China; 3 Department of Clinical Medicine, School of Medicine, Hangzhou City University, Hangzhou, China; 4 Department of Nursing, Quzhou People's Hospital, The Quzhou Affiliated Hospital, Wenzhou Medical University, Quzhou, China

**Keywords:** flexible ureteroscopic lithotripsy, hydronephrosis, renal rupture, selective renal artery embolization, urinary extravasation

## Abstract

A retrospective analysis was conducted on 4 patients who developed renal rupture and urinary extravasation after retrograde intrarenal surgery (RIRS) in our hospital. Their clinical data, intraoperative findings, rescue procedures, and outcomes were reviewed, alongside a narrative literature review of relevant studies. For the literature review, we searched PubMed and CNKI databases using the terms “RIRS,” “renal rupture,” “urinary extravasation,” “intrarenal pressure,” and “ureteroscopic complications,” without applying strict systematic selection criteria. Because these cases were collected over an extended period without a dedicated procedural registry, we did not attempt to calculate the overall incidence of this complication at our institution; rather, this series focuses on the clinical presentation, risk factors, and management strategies. We acknowledge that the small sample size, retrospective single-center design, and absence of a control group are inherent limitations of this case series. All 4 cases were successfully managed without mortality. The primary contributing factors included pre-existing moderate-to-severe hydronephrosis with thinning of the renal parenchyma (*n* = 4), duplicated kidney (*n* = 1), prior renal surgery (*n* = 2), prolonged operative time (2.5 to 3.7 h), presumed elevated intrarenal pressure (inferred from poor outflow and prolonged operative time), and pre-existing urinary tract infection (*n* = 1). Emergency interventions comprised broad-spectrum antibiotics, fluid resuscitation, percutaneous drainage of ascites (1,000–1,200 mL drained within 30 min in relevant cases), and selective renal artery embolization for active bleeding (*n* = 1). All patients recovered and were discharged; follow-up imaging showed gradual resolution of perinephric hematoma and extravasation. Renal rupture with massive urinary extravasation is a rare but severe complication of RIRS. Key preventive measures, as suggested by the published literature, may include strict case selection, adequate preoperative infection control, preoperative ureteral stenting (generally for 7–14 days according to prior reports), efforts to keep intrarenal pressure below the commonly cited threshold of 30 mmHg, and limiting operative time to the generally recommended 60–90 min, alongside early intraoperative or postoperative detection via ultrasound or CT. This series underscores that early recognition, multidisciplinary collaboration, and timely intervention, including prompt drainage, intensive care support, and selective arterial embolization when indicated, are critical for successful management.

## 1 Introduction

Retrograde intrarenal surgery (RIRS) with holmium laser lithotripsy has emerged as a cornerstone in the minimally invasive management of upper urinary tract calculi, offering advantages in terms of efficacy and patient recovery compared to percutaneous nephrolithotomy for appropriately selected stones (1). The global burden of urolithiasis continues to rise, with prevalence rates varying geographically, ranging from approximately 6% in northern China to over 10% in certain sub-Saharan African populations, underscoring the growing demand for safe and effective endoscopic interventions (2, 3). Despite its favorable safety profile, RIRS is not without risk. While major complications are relatively uncommon in large multicenter registries, with intraoperative bleeding requiring transfusion reported in 0.1% and sepsis necessitating intensive care in 1.3% of cases, reatening hemorrhage can and do occur (4). Among these, renal rupture with massive urinary extravasation is an exceptionally rare but potentially life-threatening complication that carries significant morbidity, prolonged hospitalization, increased healthcare costs, and potential mortality (5, 6).

The pathophysiology of RIRS-induced renal rupture is multifactorial, with elevated intrarenal pressure (IRP) emerging as a critical mechanistic driver. Normal physiological IRP in an unobstructed kidney ranges from 0 to 15 mmHg, but during RIRS, pressures frequently exceed dangerous thresholds, leading to pyelorenal backflow, forniceal rupture, and parenchymal injury (7). When sustained or severe, elevated IRP, often compounded by prolonged operative time, high irrigation flow rates, pre-existing hydronephrosis, and anatomical anomalies, has been associated with frank renal rupture with associated hemorrhage and urinary extravasation in the literature. We acknowledge that IRP was not directly measured in our series, and thus its role in our cases is inferred from intraoperative surrogates. Notably, patients with chronic hydronephrosis and thinned renal parenchyma are particularly vulnerable, as their renal structural integrity is already compromised (2, 7). Compounding this mechanical risk, pre-existing or procedure-associated urinary tract infection amplifies the likelihood of severe outcomes. Inflammatory changes render renal tissues more friable, while the systemic inflammatory response can progress to urosepsis, as exemplified by reported fatal cases following RIRS (5, 6). The convergence of elevated IRP, prolonged surgery, anatomical predisposition, and infection creates a “perfect storm” scenario that demands heightened clinical awareness and proactive prevention.

To date, the literature on RIRS-induced renal rupture has been largely limited to isolated case reports and small series, with few comprehensive analyses addressing the interplay of risk factors, diagnostic strategies, and multidisciplinary management approaches (6, 8). Recognizing this gap, we present a case series of four patients who developed renal rupture with massive urinary extravasation following RIRS at our institution, systematically analyzing the contributing etiologies, diagnostic pathways, and therapeutic interventions. Through this analysis and a narrative review of the contemporary literature, we aim to provide practical strategies for prevention, early recognition, and optimal management of this rare but devastating complication.

### 1.1 Case presentation

#### 1.1.1 Case 1

A 70-year-old male was admitted with a one-year history of left renal calculi and intermittent gross hematuria for four months. Physical examination on admission revealed the following: oral temperature, 36.0 °C; pulse, 66 beats/min; respiratory rate, 20 breaths/min; and blood pressure, 135/85 mmHg. The patient was alert, with no scleral icterus or palpable superficial lymphadenopathy. Cardiopulmonary auscultation was unremarkable. The abdomen was soft, without tenderness or rebound pain, and renal angle tenderness was absent. Preoperative imaging, including urinary ultrasonography and non-contrast computed tomography (CT) at our institution, demonstrated left renal calculi with moderate-to-severe hydronephrosis and a parapelvic cyst (Figure 1). The initial diagnosis was left renal calculi with hydronephrosis and a left parapelvic cyst. On Day 2, the patient underwent left ureteroscopic double-J (D-J) stent placement under intravenous anesthesia. A plain kidney-ureter-bladder (KUB) radiograph obtained on Day 3 showed radiopaque stones in the upper pole of the left kidney, with post-procedural changes of left D-J stent placement. Postoperatively, the patient experienced urinary urgency, frequency, and dysuria, along with intermittent hematuria. He was readmitted on Day 15 with a diagnosis of left renal calculi, status post left ureteral stent placement, left parapelvic cyst, and urinary tract infection. Antibiotic therapy with piperacillin-tazobactam (2.75 g intravenously every 8 h) was initiated. On Day 17, flexible ureteroscopic holmium laser lithotripsy for left renal calculi was performed using a 7.5Fr flexible ureteroscope with a 10/12Fr urinary catheter for flexible ureteroscopy, under gravity-fed irrigation. Intraoperatively, a hard stone measuring approximately 1.5 × 1.2 cm was identified in the left upper calyx. Holmium laser lithotripsy was performed at 1.0 J and 10 Hz, and fragmented stones were retrieved using a zero-tip nitinol basket. Total operative time was 3 h and 42 min. During emergence from anesthesia, the patient developed hypoxemia; the anesthesiologist noted marked abdominal distension. Bedside ultrasound revealed a hyperdense shadow and fluid accumulation around the left kidney, as well as massive intra-abdominal fluid. Immediate right lower quadrant puncture drainage yielded 1,000 mL of light red fluid. Post-intervention, the patient received antibiotic and hemostatic therapy, recovered uneventfully, and was discharged one week later. The left D-J stent was removed after two weeks, and a follow-up ultrasound performed six months post-discharge showed normal-sized kidneys with no dilation of the renal pelvis or ureter. After the surgery, the creatinine clearance rate (CCR) was 80.21 mL/min, identical to the value recorded at one day before the surgery (Supplementary Table 1).

**Figure 1:**
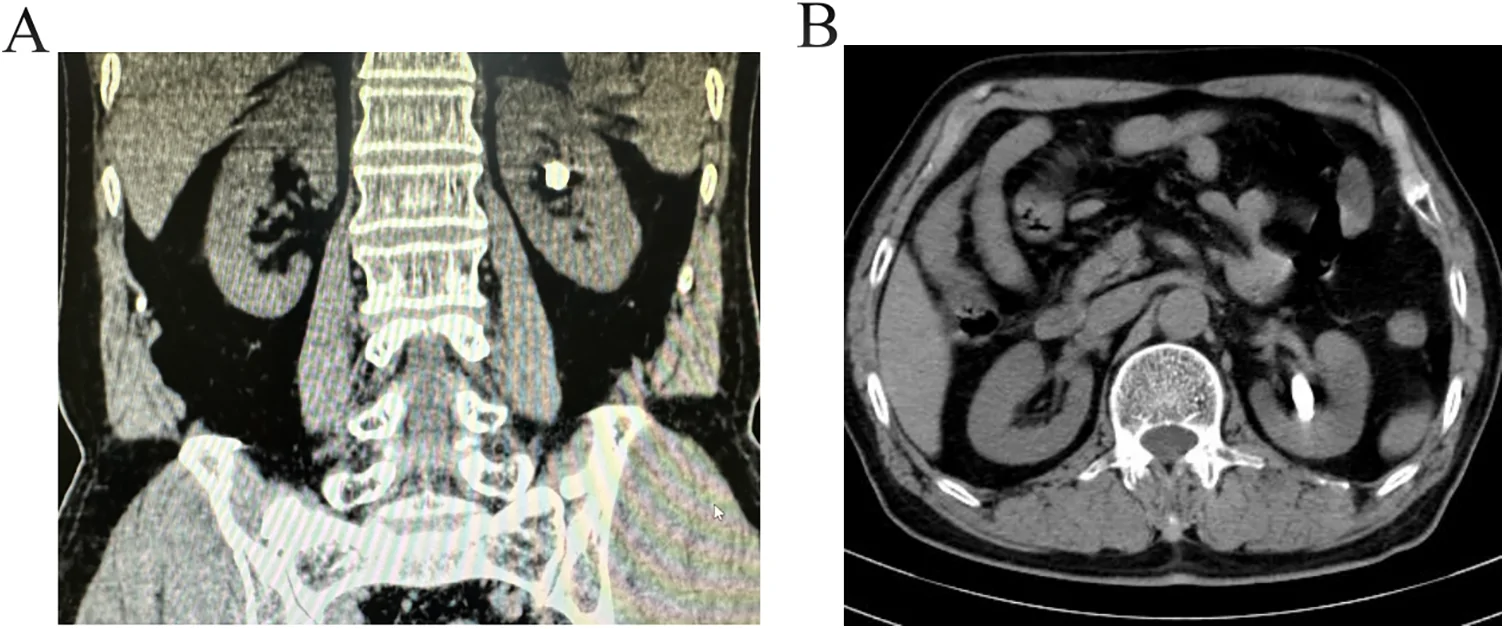
A 70-year-old male patient with a left kidney stone (case 1). Preoperative CT coronal scan **(A)** and preoperative CT sagittal scan **(B).**

#### 1.1.2 Case 2

A 22-year-old female patient was admitted to our hospital, presenting with a three-year history of recurrent dull pain in the right lumbar region, which had recurred for three days. Her medical history was notable for a cesarean section performed nine months prior. On admission, physical examination revealed the following vital signs: oral temperature 37.2 °C, pulse rate 83 beats/min, respiratory rate 16 breaths/min, and blood pressure 115/72 mmHg. No palpable superficial lymphadenopathy was detected. Pulmonary auscultation revealed clear breath sounds bilaterally without rales or rhonchi. Cardiac examination demonstrated regular rhythm without pathological murmurs. The abdomen was soft, non-tender, and without rebound tenderness or guarding; no abdominal masses were palpable. There was no percussion pain in the left renal region, whereas percussion pain was positive in the right renal region. Preoperative imaging, including ultrasonography and non-contrast CT performed at our institution on Day 1, demonstrated multiple calculi in the right kidney and renal pelvis, with marked hydronephrosis and pelvicalyceal dilatation, as well as thinning of the renal parenchyma (Figure 2). No radiopaque calculi were identified in the left kidney, ureter, or urinary bladder. Urinalysis revealed leukocyturia (WBC ++). A preliminary diagnosis of right multiple nephrolithiasis with severe hydronephrosis was established. On Day 4, the patient underwent right ureteroscopic D-J stent placement under intravenous anesthesia. A plain abdominal radiograph (KUB) obtained on Day 7, showed multiple radiopaque calculi in the right kidney, with postoperative changes consistent with right-sided D-J stent placement. The patient experienced intermittent hematuria following the procedure.

**Figure 2:**
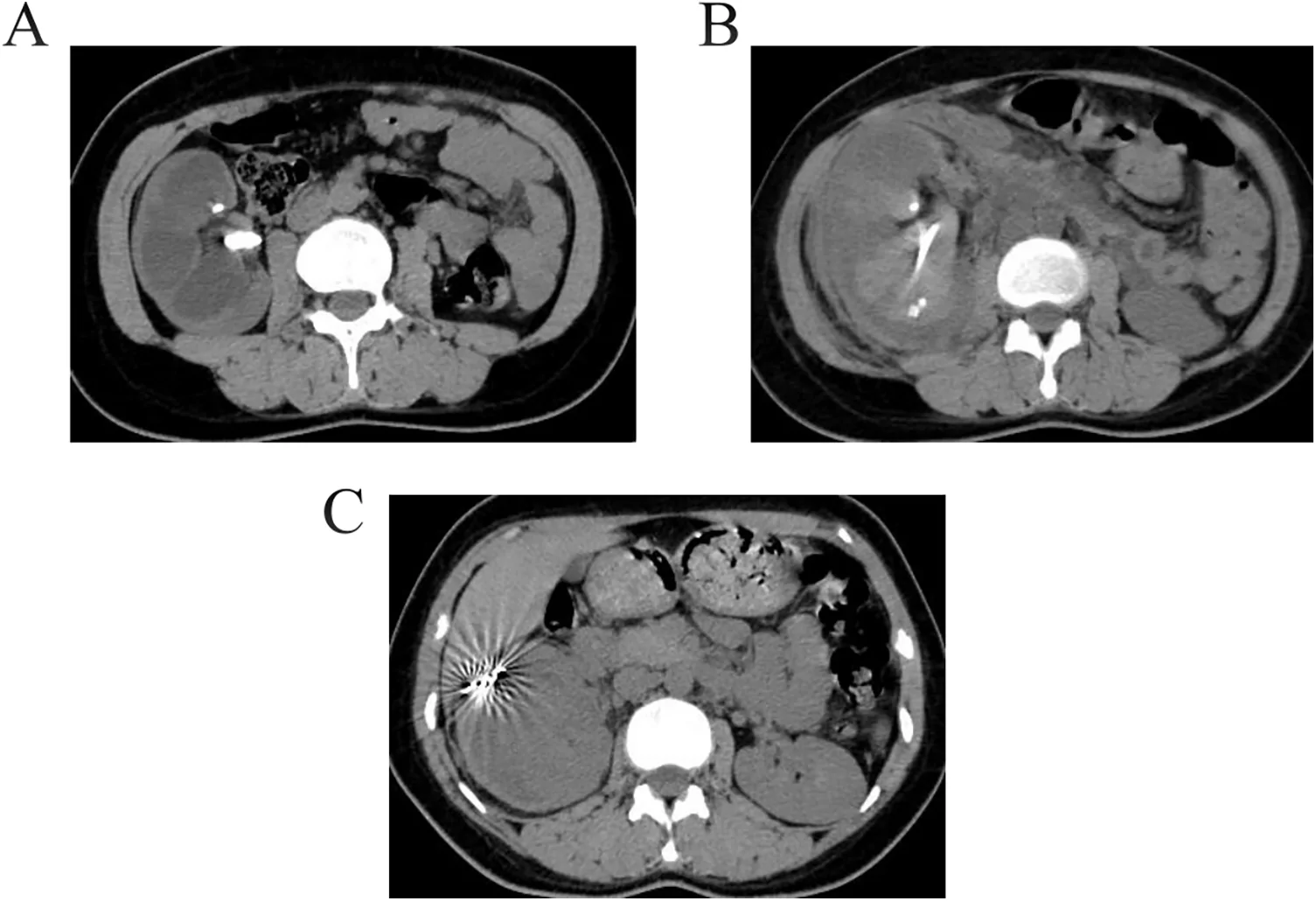
Preoperative **(A)**, postoperative day **(B)**, and postoperative half-month **(C)** CT plain-scan cross-sectional images of the 22-year-old female patient who underwent ureteroscopic lithotripsy and placement of a D-J stent (case 2).

The patient was readmitted on Day 23, with a diagnosis of right multiple nephrolithiasis with severe hydronephrosis, post-right ureteral stent placement status, and urinary tract infection. Intravenous piperacillin-tazobactam (2.75 g q8h) was administered for antibiotic therapy. On Day 25, the patient underwent RIRS with holmium laser lithotripsy using a 7.5Fr flexible ureteroscope with a 10/12Fr urinary catheter for flexible ureteroscopy, with gravity-fed irrigation. Intraoperative findings revealed a Y-shaped bifurcation of the right ureter, with two distinct ureteral branches draining separate renal moieties. The previously placed D-J stent was correctly positioned within the orthotopic renal pelvis, which contained no calculi. In contrast, the duplicated renal moiety contained an approximately 1.8 × 1.0 cm calculus, with upstream pelvicalyceal dilatation and turbid urine containing flocculent material. Holmium laser lithotripsy was performed at 0.8 J and 12 Hz, and a portion of the larger fragmented calculi was extracted using a zero-tip nitinol stone retrieval basket. A D-J stent was placed at the conclusion of the procedure. Total operative time was 3 h.

Postoperatively, the patient developed chills, high fever, and lethargy. Vital signs showed tachycardia (104 beats/min) and blood pressure of 114/75 mmHg, with gross hematuria. Emergency laboratory evaluation revealed leukopenia (white blood cell count 1.0 × 10⁹/L), neutropenia (50.0%), anemia (hemoglobin 92 g/L), and thrombocytopenia (platelet count 84 × 10⁹/L). As blood pressure progressively declined, a diagnosis of postoperative septic shock was suspected. Antibiotic therapy was switched from piperacillin-tazobactam to meropenem (1.0 g q8h), and blood cultures were obtained. Intravenous dexamethasone (10 mg) and albumin (10 g) were administered, with close monitoring of vital signs and urine output. Despite aggressive fluid resuscitation, the patient remained hypotensive and febrile by 18:30, necessitating transfer to the intensive care unit (ICU) with a diagnosis of: (1) septic shock, (2) urinary tract infection, (3) right nephrolithiasis with severe hydronephrosis, and (4) post-RIRS status. In the ICU, meropenem (1.0 g q8h) was continued. Repeat laboratory studies on October 23, 2019, showed persistent leukopenia (1.6 × 10⁹/L) with marked neutrophilia (90.8%), worsening anemia (hemoglobin 63 g/L), and progressive thrombocytopenia (platelet count 42 × 10⁹/L). Biochemical analysis revealed hypoalbuminemia (27.0 g/L). Coagulation profile demonstrated disseminated intravascular coagulation (DIC), with prolonged prothrombin time (23.3 s), reduced prothrombin activity (35.3%), international normalized ratio 1.97, prolonged activated partial thromboplastin time (53.7 s), prolonged thrombin time (19.4 s), hypofibrinogenemia (0.699 g/L), markedly elevated D-dimer (16,430 μg/L), and elevated fibrinogen degradation products (40.8 μg/mL). The patient received transfusion support including 500 mL of fresh frozen plasma, 9.5 units of cryoprecipitate, 3.5 units of packed red blood cells (type A), and 800 IU of prothrombin complex concentrate.

Non-contrast CT of the urinary tract on Day 26, revealed post-right D-J stent placement status with a large perirenal hematoma, extensive exudation, and pelvic fluid collection. Renal ultrasonography suggested a right duplicated kidney, with an intrarenal blood clot in the lower renal moiety along with multiple calculi, a 6.0 cm right perirenal hematoma, a 7.5 cm intra-abdominal hematoma in the lower abdomen, and a right-sided pleural effusion. Following multidisciplinary discussion on Day 26, at 21:08, the patient was diagnosed with: (1) septic shock, (2) hemorrhagic shock, (3) urinary tract infection, (4) right nephrolithiasis with severe hydronephrosis, (5) right renal rupture with hemorrhage, and (6) post-RIRS status. The patient was transferred to Jinhua Central Hospital, where angiography-guided super-selective renal artery embolization was performed. Intraprocedural findings confirmed active bleeding from a branch of the right renal artery, which was successfully embolized. Post-embolization management included continued antibiotic therapy and fluid resuscitation, following which the patient's condition stabilized. The patient was discharged one week later. Outpatient follow-up with non-contrast CT two weeks late, demonstrated a right perirenal hematoma, right duplicated kidney, post-D-J stent placement status, and intra-abdominal fluid collection. Repeat CT 1.5 months later, showed a right localized perirenal fluid collection (urinoma) with significant resolution of the hematoma compared to the scan 1 month before, along with persistent right nephrolithiasis and a well-positioned right D-J stent. The patient underwent uneventful right ureteroscopic D-J stent removal. At six-month follow-up, renal ultrasonography revealed a right duplicated kidney, persistent multiple right renal calculi, and a residual right perirenal hematoma measuring 3.4 × 1.2 cm. After the surgery, her CCR was significantly declined (Supplementary Table 1).

#### 1.1.3 Case 3

A 50-year-old male was admitted with a three-day history of right flank pain. Two years prior, he had undergone RIRS for right upper ureteral calculi complicated by severe hydronephrosis in our institution. Intraoperative findings at that time included a single approximately 1.2 × 1.0 cm calculus in the right upper ureter, encased by periureteral polyps, with turbid urine in the renal pelvis proximal to the obstruction, and multiple right renal calculi, the largest measuring approximately 0.8 × 0.6 cm. Holmium laser lithotripsy was performed, and larger fragments were retrieved using a stone extraction basket; however, residual lower pole calculi could not be reached by the flexible ureteroscope and thus were not treated. Additionally, the patient had a history of laparoscopic bilateral ureterolithotomy performed ten years previously. Upon admission, vital signs were: temperature 36.0 °C, heart rate 68 beats/min, respiratory rate 18 breaths/min, and blood pressure 149/76 mmHg. Physical examination was unremarkable except for mild right costovertebral angle tenderness. A non-contrast CT scan of the urinary tract (two days before this hospitalization) revealed multiple radiopaque stones at the proximal right ureter and renal pelvis, with marked pelvicalyceal dilatation, thinning of the renal parenchyma, and obvious perirenal stranding (Figure 3). A diagnosis of right proximal ureteral calculi with severe hydronephrosis and multiple right renal calculi was established. On Day 2, a right ureteral D-J stent was placed under intravenous anesthesia. A plain abdominal radiograph (KUB) the following day demonstrated beaded radiopaque densities of varying sizes in the right renal region anda stent in the right ureter. The patient experienced intermittent hematuria postoperatively.

**Figure 3:**
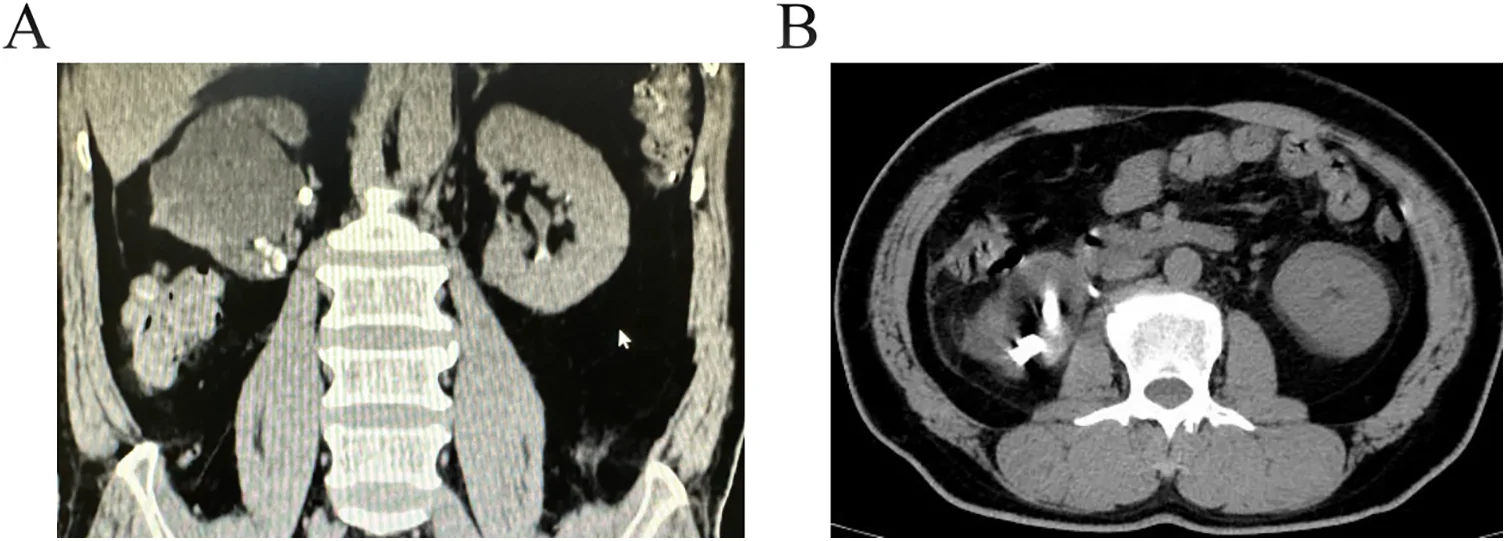
Preoperative CT coronal scan **(A)** and preoperative CT sagittal scan **(B)** of a 50-year-old male patient before ureteroscopic lithotripsy (case 3).

He was readmitted two weeks later, with a diagnosis of right proximal ureteral calculi with severe hydronephrosis, multiple right renal calculi, status post right ureteral stent placement, and urinary tract infection. On Day 2, the patient underwent right RIRS with holmium laser lithotripsy, D-J stent exchange, and exploratory laparoscopy with drainage, using a 7.5Fr flexible ureteroscope with a 10/12Fr urinary catheter for flexible ureteroscopy, with gravity-fed irrigation. The total operative time was 2.5 h. Intraoperative findings included multiple right lower pole calculi, the largest measuring 1.5 × 1.2 cm, with turbid urine. Holmium laser lithotripsy was performed, and fragments were extracted with a nitinol basket; however, the precise laser energy and frequency settings were not consistently recorded in the operative notes. After fragmentation of most calculi with the holmium laser, markedly reduced outflow through the ureteral access sheath was noted. Abdominal examination revealed distension, raising suspicion of renal rupture at a site of calculus impaction overlying thin parenchyma. The procedure was therefore terminated, and a D-J stent was placed. Intraoperative ultrasound consultation confirmed massive ascites and perirenal fluid and blood collection. A right lower quadrant drainage catheter was inserted, draining 1,200 mL of pinkish fluid within 30 min. Postoperative laboratory studies on Day 3, showed a white blood cell count of 14.4 × 10⁹/L (87.2% neutrophils), hemoglobin 142 g/L, platelet count 166 × 10⁹/L, and a rapid C-reactive protein level of 6.5 mg/L. A non-contrast CT scan of the urinary tract on the same day demonstrated a right D-J stent, pelvicalyceal dilatation with multiple calculi, a thinned renal cortex, and intrarenal and perirenal hematoma with extensive perirenal exudative changes. The patient was discharged six days later. The right ureteral D-J stent was removed after one month. A follow-up ultrasound six months post-discharge revealed multiple right renal calculi with severe hydronephrosis, along with multiple crystals in the left kidney and a left renal cyst. After the surgery, his CCR was slightly declined (Supplementary Table 1).

#### 1.1.4 Case 4

A 46-year-old male patient presented with a two-year history of dull pain in the right lumbar region and was admitted. Two years prior to admission, he had undergone left-sided percutaneous nephrolithotomy (PCNL), left-sided RIRS, and right-sided ureteroscopic lithotripsy for bilateral upper ureteral calculi complicated by hydronephrosis and renal insufficiency. Bilateral D-J stents were removed two weeks after those procedures; however, the patient continued to experience persistent dull pain in the right lumbar region. One day before, an unenhanced CT scan of the urinary tract performed in the outpatient clinic revealed a right upper ureteral calculus with hydronephrosis, right kidney atrophy, slightly decreased density of the left renal parenchyma, suspected left renal calcification, and multiple small bilateral renal calculi (Figure 4). Upon admission, physical examination findings were as follows: oral temperature 36.5 °C, pulse 68 beats per minute, respiratory rate 19 breaths per minute, blood pressure 150/86 mmHg. The patient was alert, with clear breath sounds bilaterally, regular heart rate without murmurs, soft abdomen without tenderness or rebound tenderness, and no lower extremity edema. A repeated unenhanced CT scan of the urinary tract confirmed the presence of a right upper ureteral calculus with severe hydronephrosis, right kidney atrophy, and multiple small bilateral renal calculi. The preliminary diagnoses were right upper ureteral calculus with hydronephrosis, right kidney atrophy, and multiple bilateral renal calculi. On Day 2, the patient underwent left ureteroscopic D-J stent placement under intravenous anesthesia. The subsequent kidney-ureter-bladder (KUB) radiograph on Day 3, showed a right upper ureteral radiopaque calculus with the D-J stent twisted at the lower margin of the calculus. After discharge, the patient experienced urinary urgency, frequency, dysuria, and occasional hematuria.

**Figure 4:**
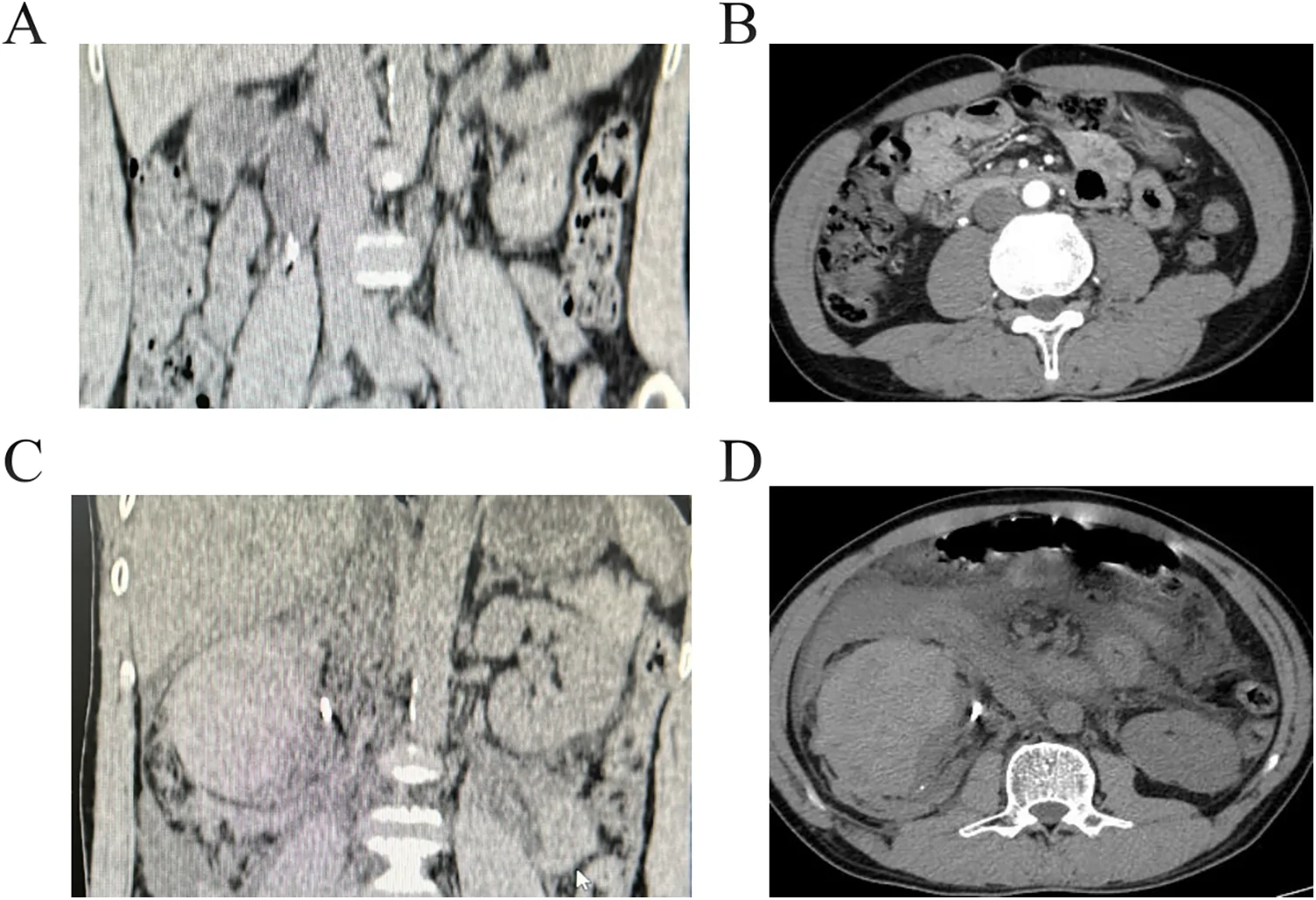
46-year-old male patient with upper segment calculi of both ureters and hydronephrosis. Preoperative CT plain scan coronal view **(A)**, preoperative CT plain scan sagittal view **(B)**, postoperative CT plain scan coronal view **(C)**, and postoperative CT plain scan sagittal view **(D)** (Case 4).

He was readmitted two weeks later, with diagnoses of right upper ureteral calculus with hydronephrosis, postoperative status of right ureteral D-J stent placement, right kidney atrophy, multiple bilateral renal calculi, and urinary tract infection. Intravenous piperacillin-tazobactam (3.375 g q8h) was administered for anti-infective therapy. On Day 3, RIRS with holmium laser lithotripsy and D-J stent exchange was performed on the right kidney and ureter using a 7.5Fr flexible ureteroscope with a 10/12Fr urinary catheter for flexible ureteroscopy, with gravity-fed irrigation. Total operative time was 3 h. Holmium laser lithotripsy was performed at 1.0 J and 8 Hz, with stone fragments retrieved using a zero-tip nitinol basket. Intraoperative findings included a tortuous and stenotic right upper ureter with multiple polyps; laser incision was required to traverse the stenosis, revealing two calculi measuring approximately 0.6 × 0.8 cm tightly encased by mucosa. Multiple intrarenal calculi measuring 3–5 mm were observed, along with marked pelvicalyceal dilatation and cloudy urine containing purulent flocculent material. Due to the tortuous and stenotic ureter, intraoperative irrigation outflow was persistently poor. Postoperatively, upon routine ward round, the patient was found to have abdominal distension. Laboratory investigations revealed a white blood cell count of 13.7 × 10⁹/L (88.5% neutrophils), hemoglobin 131 g/L, hematocrit 0.399, and platelet count 199 × 10⁹/L. Bedside ultrasound demonstrated a right perirenal hematoma and intra-abdominal fluid collection. A drainage catheter was placed in the right lower quadrant, yielding 1,050 mL of light red fluid within 30 min. Unenhanced CT of the urinary tract on Day 4, confirmed a right perirenal hematoma with an indwelling right ureteral D-J stent. Follow-up CT at 1.5 months later, showed a right perirenal hematoma, indwelling right ureteral D-J stent, and multiple small bilateral renal calculi. Repeat CT at three months later, revealed a small patchy hyperdensity in the right perirenal space, persistent indwelling right ureteral D-J stent, right kidney atrophy, and small bilateral renal calculi. The right ureteral D-J stent was then removed. Follow-up ultrasound at another 3 months later, demonstrated right kidney atrophy and small bilateral renal calculi. After the surgery, his CCR was slightly increased (Supplementary Table 1) and other parameters were listed in Supplementary Table 2.

## 2 Discussion

The widespread adoption of flexible ureteroscopic lithotripsy has fundamentally transformed the management of upper urinary tract calculi (9). However, insufficient understanding of its operational nuances and potential complications continues to precipitate adverse outcomes, as documented in the literature. Drawing from our four cases, this analysis examines the contributing factors, diagnostic pathways, and management strategies derived from our institutional experience.

## 3 Pathophysiological mechanisms of renal rupture

### 3.1 Pre-existing structural abnormalities

Our series highlights that compromised renal architecture appears to represent a critical predisposing factor. Among our four patients, one presented with moderate-to-severe hydronephrosis secondary to left renal calculi, while three exhibited severe hydronephrosis associated with ureteronephric stones, with hydronephrosis duration exceeding one year in all cases. Cases 3 and 4 had histories of multiple prior surgical interventions; Case 2 demonstrated a duplicated collecting system, with intraoperative findings revealing a stone-bearing, hydronephrotic calix devoid of a preoperatively placed double-J stent; and Case 4 displayed tortuous proximal ureteral stenosis with multiple polyps necessitating laser incision.

Moderate-to-severe hydronephrosis induces pathological changes beyond elevated baseline IRP, including renal parenchymal thinning, caliceal dilation, capsular tension, and microvascular atrophy. During RIRS, continuous saline irrigation, essential for maintaining a clear visual field and facilitating scope navigation, inevitably elevates IRP. Hydronephrotic kidneys exhibit markedly diminished capacity to withstand pressure increases compared to their non-hydronephrotic counterparts (10). This pressure-mediated augmentation of tissue permeability predisposes the renal capsule and parenchyma to rupture and hemorrhage. Furthermore, ureteral stricturotomy or inadvertent sheath-induced tears may perforate under high-pressure conditions. Consistent with our observations, Cui et al. identified moderate-to-severe hydronephrosis as a predominant predisposing condition for RIRS-induced renal rupture and hemorrhage. Case 4, presenting with an atrophic kidney, the end-stage consequence of chronic hydronephrosis (11), demonstrated exceptionally poor tolerance to further IRP elevation and subsequently developed renal rupture.

In addition to hydronephrosis, other anatomical and historical factors in our series contributed to the complication. Case 2 had a duplicated collecting system, which made complete preoperative drainage difficult. Cases 3 and 4 had a history of multiple prior procedures, which induced progressive structural alterations that further compounded the risk. These observations are consistent with previous reports highlighting the vulnerability of anomalous kidneys and surgically altered upper tracts (12–15).

### 3.2 Intrarenal pressure elevation during RIRS

The fundamental mechanical conflict inherent to RIRS is that while a clear operative field necessitates continuous irrigation, this same irrigation inevitably increases IRP. The renal fornix represents the region of greatest structural vulnerability within the pyelocaliceal system; when IRP exceeds 75 mmHg (1 mmHg = 0.133 kPa) as reported in physiological studies, renal rupture may occur (16). Multiple intraoperative factors contribute to pathologic IRP elevation: sustained irrigation flow required for visualization; repeated advancement and withdrawal of the ureteroscope; the shockwave effect of holmium laser lithotripsy, particularly at high-energy settings; and compromised irrigation outflow due to ureteral strictures or polyps that constrict the scope. Absence of preoperative ureteral catheterization, failure to place or properly position a ureteral access sheath (UAS), and poor outflow during the procedure all predict elevated IRP.

Under physiological conditions, baseline human IRP approximates 7.35 mmHg (17). As IRP rises above 30 mmHg, pathological sequelae ensue: initial pyelovenous and pyelolymphatic backflow with disruption of basement membrane integrity; subsequent subcapsular and sinus extravasation; and, with continued pressure escalation, mucosal disruption, hemorrhagic foci, and forniceal rupture culminating in urinary extravasation into the peritoneal or thoracic cavities (16, 18). In the presence of pre-existing renal pathology, this cascade is both accelerated and amplified, as our cases demonstrate. However, we emphasize that intrarenal pressure was not directly measured during any of the procedures; therefore, the role of elevated IRP in our cases remains an inference based on intraoperative surrogates, including prolonged poor outflow, anatomical obstruction, and extended operative time, rather than a directly established causative factor.

### 3.3 Stone burden, location, and prolonged operative duration

Stone composition and total burden directly govern procedural complexity and operative time. For large calculi (maximum diameter >2.0 cm) or stones of high density, staged procedures are recommended to reduce complication rates (19, 20). A narrow infundibulopelvic angle (IPA) poses particular challenges for flexible ureteroscopic access to lower-pole calculi, as the limited intrarenal working space necessitates extreme scope deflection that may be obstructed at the caliceal infundibulum. Under conditions of unfavorable IPA combined with high stone density (as measured by Hounsfield units), a staged approach is often required for complete stone clearance (21). Current guidelines recommend operative durations of 60–90 min (22, 23). In our series, Cases 1, 2, and 4 exceeded three hours, while Case 3 required 2.5 h, each substantially exceeding recommended time limits. Specifically, Case 2 involved a Y-type bifid ureter with a duplicated collecting system; although a double-J stent had been placed preoperatively, the surgery still proceeded for three hours due to the complex anatomy and the inability to place a UAS, which likely contributed to poor intraoperative outflow and elevated intrarenal pressure. In Case 3, although the initial procedure had already identified lower-pole stones beyond the reach of the flexible ureteroscope, the second procedure nevertheless expended 2.5 h attempting to access and fragment these stones. Case 4 required three hours due to complex anatomy including tortuous proximal ureteral stenosis with multiple polyps requiring laser incision, coupled with poor intraoperative outflow. Finally, Case 1, despite non-complex stone size and location, required 3 h and 42 min attributable to the operator's early learning curve and lack of procedural fluency.

### 3.4 Infection as a contributing and exacerbating factor

In our series, Case 2 exhibited preoperative pyuria (WBC ++ on urinalysis), indicating established urinary tract infection that was not adequately addressed prior to intervention. Urinary tract and perinephric infections induce local tissue edema and friability; under elevated IRP, these inflamed tissues are prone to disruption, culminating in renal parenchymal and vascular rupture. Such cases frequently present with early signs of septic shock, including extreme leukocytosis or leukopenia (24), findings that were evident in Case 2.

## 4 Diagnosis and acute management

The clinical presentation of renal rupture with urinary extravasation varies according to timing of onset. Intraoperative manifestations include prolonged poor or absent outflow from the UAS, desaturation on pulse oximetry, and tachycardia. Upon suspecting renal rupture, immediate abdominal examination for distension and rigidity, together with bedside ultrasonography to evaluate for intra-abdominal or intrathoracic fluid collections, perinephric fluid, and parenchymal or perinephric hematoma, is mandatory. Stat laboratory studies should include complete blood count, blood gas analysis, and electrolytes. In the absence of abnormalities and with stable hemodynamics, the procedure may continue; otherwise, double-J stent placement, prompt termination of the procedure, percutaneous drainage of ascites, and resuscitation with blood products and intravenous fluids are indicated.

Postoperative presentations predominantly feature persistent flank or abdominal pain, which may mimic obstructive symptoms attributable to the double-J stent or an acute surgical abdomen (25). Diagnosis is confirmed through serial hemoglobin measurements and imaging; bedside ultrasonography represents the initial modality of choice due to its availability and safety profile. Contrast-enhanced CT of the urinary tract provides superior delineation of hemorrhage extent and localization, as well as quantification of extravasated urine, thereby guiding subsequent intervention (26). However, CT requires patient transport, which may exacerbate active bleeding and should therefore be undertaken with appropriate precautions.

For management of established renal rupture with massive urinary extravasation, conservative measures, including broad-spectrum antibiotics, hemodynamic support, strict bed rest, and serial monitoring of hemoglobin and hematoma size, achieve successful outcomes in most patients. Renal hematomas typically resorb spontaneously over time. In refractory cases, selective renal arterial embolization (digital subtraction angiography-guided) is the preferred intervention (27); open surgical exploration with repair or nephrectomy is generally discouraged except in hemodynamically unstable patients failing endovascular salvage.

## 5 Preventive strategies

### 5.1 Preoperative optimization

#### 5.1.1 Infection control

A history of recent stone-related infection correlates strongly with postoperative infectious complications; therefore, preoperative sterilization of the urinary tract is paramount (28). Appropriate measures include: (1) Collection of properly obtained urine specimens; if urinalysis reveals pyuria or bacteriuria, culture-directed antibiotic therapy is recommended for 2–3 days until normalization is confirmed. Preoperative culture- and sensitivity-guided antibiotic therapy for a full course (minimum 3 days) significantly reduces the incidence of post-percutaneous nephrolithotomy systemic inflammatory response syndrome (SIRS) (29). (2) Prophylactic antibiotic administration (30, 31). For many patients with upper tract calculi, even those without fever, pyuria, or bacteriuria, broad-spectrum antibiotics (primarily targeting gram-negative bacilli) substantially reduce the risk of urosepsis. Some reports suggest that seven days of preoperative nitrofurantoin effectively prevents urosepsis (32, 33).

#### 5.1.2 Anatomical risk stratification and contingency planning

When preoperative imaging identifies unfavorable anatomy, including narrow IPA, severe hydronephrosis, renal malrotation, or duplicated collecting systems, the procedure may be best performed by an experienced surgical team, if available. Intraoperative strategies should prioritize IRP minimization and abbreviated operative time, with consideration of staged procedures when indicated. Patients must be thoroughly counseled preoperatively regarding the elevated technical difficulty and attendant risks.

#### 5.1.3 Pre-stenting for 7–14 days

All four patients in our series underwent preoperative double-J stent placement. Pre-stenting for more than the commonly recommended 7–14 days facilitates subsequent UAS insertion during RIRS; a larger-caliber sheath significantly reduces IRP as reported in previous studies (34). Approximately 10% of flexible ureteroscopic procedures are complicated by inability to negotiate a narrow ureteral lumen (35). Zhang et al. reported 100% successful UAS placement in 12 patients, which they attributed to pre-stenting performed two weeks prior to lithotripsy (36).

### 5.2 Intraoperative pressure control, time limitation, and double-J stenting

#### 5.2.1 Pressure management

The direct relationship between intraoperative IRP and the risk of renal rupture with urinary extravasation is well established in the physiological and endourological literature. A consensus threshold of 30 mmHg for pyelorenal backflow has been endorsed internationally based on physiological studies and clinical experience, and clinical practice should aim to maintain IRP below this limit when feasible (37). The UAS outflow should be monitored continuously; any reduction in outflow warrants prompt investigation and corrective action. No circumstance justifies continued prolonged lasering in the absence of adequate outflow.

#### 5.2.2 Time limitation

Operative duration is generally recommended to be limited to 60–90 min, a timeframe that has been recommended in the literature to balance efficacy and safety. These principles are well aligned with contemporary international guidelines. The EAU Guidelines on Urolithiasis recommend limiting RIRS operative time to below 90 min (https://uroweb.org/guidelines/urolithiasis). The AUA Guidelines (2026) similarly emphasize that prolonged operative time increases the risk of infectious and hemorrhagic complications, and suggest that urologists should maintain a low threshold for terminating the procedure and staging the treatment (38). Regarding intrarenal pressure, both guidelines acknowledge that pressures exceeding 30 mmHg are associated with pyelorenal backflow and increased complication rates, and recommend measures such as ureteral access sheath placement and gravity-fed irrigation to minimize pressure elevation. In our series, the absence of routine intrarenal pressure monitoring and the failure to strictly adhere to these time limits contributed to the observed complications, and we have since modified our institutional protocol to comply with these guideline recommendations. For large or dense stones, staged procedures are strongly preferred. The pursuit of complete stone clearance in a single session must never compromise patient safety. We acknowledge that in the present series, the decision to proceed with a single prolonged RIRS session rather than a staged approach or alternative procedures such as percutaneous nephrolithotomy (PCNL) or shockwave lithotripsy warrants critical reflection. In Cases 1, 2, and 4, the operative time exceeded three hours despite the presence of severe hydronephrosis, thinned parenchyma, or complex anatomy (duplicated collecting system in Case 2, and tortuous proximal ureteral stenosis with polyps in Case 4). These decisions were influenced by several factors: first, the preoperative imaging did not consistently alert the surgical team to the full extent of anatomical complexity; second, patient preference strongly favored the minimally invasive nature of RIRS over PCNL; and third, the surgeon at the time believed that complete stone clearance could be achieved in a single session. In retrospect, earlier termination of the procedure with placement of a double-J stent, followed by a planned second-stage RIRS or conversion to PCNL for the residual stones, would have been a safer strategy. This experience has since modified our institutional practice, and we now adopt a lower threshold for staged procedures when unfavorable anatomy or poor intraoperative outflow is encountered.

#### 5.2.3 Double-J stenting

A double-J stent is generally recommended at procedure conclusion unless contraindicated. Stenting facilitates urinary drainage, reduces postoperative IRP, and promotes recovery in cases where intraoperative renal rupture or urinary extravasation occurs.

#### 5.2.4 Intraoperative monitoring

Based on our experience, we recommend that for procedures exceeding one hour, stat laboratory tests (complete blood count, C-reactive protein) should be obtained. Significant hemoglobin decline or extreme leukocyte abnormalities should prompt immediate ultrasonography to rule out hematoma or urinary extravasation. In our series, Cases 1 and 3 were diagnosed intraoperatively by ultrasound and managed with immediate percutaneous drainage, which proved effective. We suggest that when the procedure proves excessively difficult, the operator consider terminating early and place a double-J stent rather than persisting with prolonged attempts.

### 5.3 Postoperative protocol

Within one hour postoperatively, complete blood count, CRP, procalcitonin, electrolytes, and albumin are recommended to be obtained. When renal rupture with urinary extravasation is identified, the emergency protocol described above should be activated, and transfer to an intensive care unit should be considered. In our series, Cases 2 and 4 were diagnosed postoperatively on the ward and managed emergently. As soon as clinically feasible, non-contrast CT of the urinary tract should be performed. CT provides precise characterization of hemorrhage extent and localization, as well as quantification of urinary extravasation, thereby guiding subsequent management. Drains placed for urinary extravasation may be removed 1–2 days after drainage ceases. Patients with renal rupture, once stabilized, should avoid vigorous activity for four weeks and should undergo scheduled follow-up. We acknowledge that many of the preventive and management recommendations outlined above are derived from our institutional experience and expert interpretation of the published literature, rather than from high-level evidence generated by this small case series. Where applicable, we have cited contemporary guidelines to support recommendations that are broadly accepted in the field; however, the strength of these recommendations should be interpreted in light of the inherent limitations of our study design. Based on our institutional experience and the principles discussed above, we propose a simple clinical algorithm for the diagnosis and management of renal rupture with urinary extravasation following RIRS, as summarized in Supplementary Figure 1.

We acknowledge that the present findings must be interpreted in the context of several important limitations. First, the retrospective and single-center design, combined with the very small sample size (four cases) and the absence of a control or comparison group, precludes any determination of incidence, independent risk factors, or causal relationships; our observations represent associations rather than causations. Second, potential selection bias cannot be excluded, as the four cases were identified retrospectively over an extended period without a dedicated procedural registry. Third, the heterogeneity among the four cases varying in anatomy, stone burden, prior surgical history, and clinical presentation, limits the generalizability of our findings. Fourth, the lack of direct intrarenal pressure monitoring means that elevated IRP is inferred from intraoperative surrogates (poor outflow, anatomical obstruction, and prolonged operative time) and physiological principles, rather than confirmed by measured data. Fifth, the heterogeneous retrieval of operative parameters, such as irrigation pressure and laser settings, reduced the granularity of our technical evaluations, though a consistent operative setup provides a stable framework. Sixth, the unavailability of differential renal scans precluded any assessment of split renal function. We did not conduct long-term follow-up of renal function yet. Finally, the accompanying literature review was conducted as a narrative review rather than a systematic synthesis, which limits the strength of the evidence base informing our recommendations. Many of the preventive and management strategies outlined are derived from our institutional experience and expert interpretation, rather than from high-level evidence generated by this case series. Notwithstanding these methodological constraints, we believe that the detailed clinical documentation of these rare adverse events offers significant educational value and practical guidance for managing similar cases in future urological practice.

## 6 Conclusion

In summary, RIRS, while an effective and increasingly utilized modality for upper tract stone disease, carries a rare but devastating risk of renal rupture with hemorrhage and massive urinary extravasation. Meticulous preoperative patient selection and optimization, attention to published principles of intraoperative IRP control and time limitation, and systematic postoperative surveillance may help mitigate this complication. The four cases presented herein underscore that anatomical anomalies, chronic hydronephrosis, prolonged operative duration, presumed uncontrolled IRP (inferred from poor outflow and prolonged procedures, rather than directly measured), and pre-existing infection should be regarded as potential contributing factors that may be associated with renal rupture in a multifactorial manner; addressing each component is necessary to achieve safe outcomes. Most importantly, our experience reinforces that early recognition, multidisciplinary management, and timely intervention are the cornerstones of optimizing outcomes in this rare but devastating complication. These observations, however, are derived from a small case series and should be interpreted with caution, as they do not establish causal relationships.

## Data Availability

The original contributions presented in the study are included in the article/Supplementary Material, further inquiries can be directed to the corresponding author.

## Supplementary material

Supplementary Figure 1 Clinical algorithm for the management of renal rupture with urinary extravasation following flexible ureteroscopic lithotripsy (RIRS).
