# Supplementary material for: Case Report: Renal rupture and massive urine extravasation following flexible ureteroscopic lithotripsy: analysis of 4 cases and literature review
Source: Front Med (Lausanne). 2026 Sep 29;13:1914419. doi: 10.3389/fmed.2026.1914419 (PMC13657939; doi:10.3389/fmed.2026.1914419)
Supplement: Supplementary file 1 [file Datasheet1.docx]

**Supplementary Table 1**. Available renal function for all four cases before and after the surgery.

| **Cases** | **Sex70** | **Age** | **Weight (kg)** | **Serum creatinine (μmol/L)** | | **Creatinine clearance rate (CCR, mL/min)** | |
| --- | --- | --- | --- | --- | --- | --- | --- |
|  |  |  |  | Before the surgery | After the surgery | Before the surgery | After the surgery |
| Case 1 | Male | 70 | 70 | 75 | 75 | 80.21 (1 day before) | 80.21 (1 day after) |
| Case 2 | Female | 22 | 53 | 47 | 123 | 138.87 (1 day before) | 53.06 (1 day after) |
| Case 3 | Male | 50 | 69 | 68 | 72 | 112.13 (1 day before) | 105.90 (1 day after) |
| Case 4 | Male | 46 | 50 | 123 | 122 | 46.92 (2 day before) | 47.30 (1 day after) |
