# Supplementary material for: Case Report: Renal rupture and massive urine extravasation following flexible ureteroscopic lithotripsy: analysis of 4 cases and literature review
Source: Front Med (Lausanne). 2026 Sep 29;13:1914419. doi: 10.3389/fmed.2026.1914419 (PMC13657939; doi:10.3389/fmed.2026.1914419)
Supplement: Supplementary file 2 [file Datasheet2.docx]

**Supplementary Table 2**. Summary of clinical characteristics, intraoperative findings, management, and outcomes of four patients with renal rupture following RIRS.

| **Characteristic** | **Case 1** | **Case 2** | **Case 3** | **Case 4** |
| --- | --- | --- | --- | --- |
| Age (years) / Sex | 70 / Male | 22 / Female | 50 / Male | 46 / Male |
| Stone burden / location | 1.5 × 1.2 cm / left upper calyx | 1.8 × 1.0 cm / duplicated right moiety | 1.5 × 1.2 cm / right lower pole | 0.6 × 0.8 cm ×2 / right upper ureter |
| Degree of hydronephrosis | Moderate-to-severe | Severe | Severe | Severe (with right kidney atrophy) |
| Anatomical abnormalities | Parapelvic cyst | Duplicated right ureter (Y-type) | None identified | Tortuous proximal ureteral stenosis with polyps |
| Previous interventions | None | Cesarean section (9 months prior) | Prior RIRS (2 years), laparoscopic bilateral ureterolithotomy (10 years) | Bilateral PCNL, RIRS, ureteroscopic lithotripsy (2 years prior) |
| Evidence of infection | UTI (readmission) | Pyuria (WBC ++), septic shock | UTI (readmission) | UTI (readmission), purulent urine intraop |
| Pre-stenting | Yes (D-J, 15 days before RIRS) | Yes (D-J, 21 days before RIRS) | Yes (D-J, 14 days before RIRS) | Yes (D-J, 14 days before RIRS) |
| Operative duration | 3 h 42 min | 3 h | 2.5 h | 3 h |
| Intraoperative outflow / UAS | Not specified | Poor outflow (no UAS placed) | Markedly reduced outflow | Persistently poor outflow (tortuous ureter) |
| Clinical presentation of rupture | Hypoxemia, abdominal distension (emergence) | Septic shock, hemorrhagic shock (postop) | Abdominal distension (intraop) | Abdominal distension (postop) |
| Imaging findings | Perirenal fluid, massive ascites | Perirenal hematoma, intra-abdominal hematoma, pleural effusion | Perirenal hematoma, ascites | Perirenal hematoma, intra-abdominal fluid |
| Rescue treatment | Percutaneous drainage (1,000 mL) | ICU, embolization, transfusion, meropenem | Percutaneous drainage (1,200 mL) | Percutaneous drainage (1,050 mL) |
| Outcome | Discharged day 7; stent removed at 2 weeks | Discharged day 7; residual hematoma at 6 months | Discharged day 6; stent removed at 1 month | Discharged; stent removed at 3 months |

Note: UAS, ureteral access sheath; UTI, urinary tract infection; ICU, intensive care unit; PCNL, percutaneous nephrolithotomy; RIRS, retrograde intrarenal surgery; D-J, double-J.
